# Giant lipoma of the third finger of the hand

**DOI:** 10.1186/2193-1801-2-164

**Published:** 2013-04-16

**Authors:** Luis Ramirez-Montaño, Ricardo Pacheco Lopez, Nicolas Sastre Ortiz

**Affiliations:** Plastic and Reconstructive Surgery, General Hospital of Mexico, Eje 2A Sur (Dr. Balmis) 148 Doctores, Cuauhtémoc México City, 0672 Mexico

## Abstract

We report a case of a 50-year old female presenting with a giant tumor on the volar aspect of the third finger of the left hand, a thorough clinical and paraclinical evaluation followed by surgical resection resulted in a benign lipoma with an uneventful postoperative course. We present this case due to its rare location and repercussion in the decision making process when other more common similar pathologies with varying prognosis are conceived.

## Introduction

Excluding cutaneous malignancy, 95% of tumors of the hand are of benign origin. Non-neoplastic ganglions are probably the most common on hand and wrist. After these tumors, inclusion cysts, warts, giant cell tumors, granulomas and hemangiomas follow in frequency. Lipomas are benign mesenchymal neoplasms occurring in areas of abundant adipose tissue. They are not very common in the hand, when present, they predominate in the thenar and hypothenar regions (Al Qattan et al. 2005). Those involving the fingers are very rare, with an incidence of 1% (De Giorgi et al. 2005). The clinical spectrum varies depending on its location, presenting as a painless slow growing mass, that affects the mobility of the finger due to its size, they may also cause neurologic changes in the peripheral nerves of the hand (Leffert 1972). Stein reported the first lipoma of the finger in 1959 (Stein 1959), afterwards, 14 similar cases were identified in the literature (Ersozlu et al. 2007). We present a case of a giant lipoma in the third finger of the dominant hand, with no prior traumatic history of the proximal and middle phalanx.

## Case report

**Figure 1:**
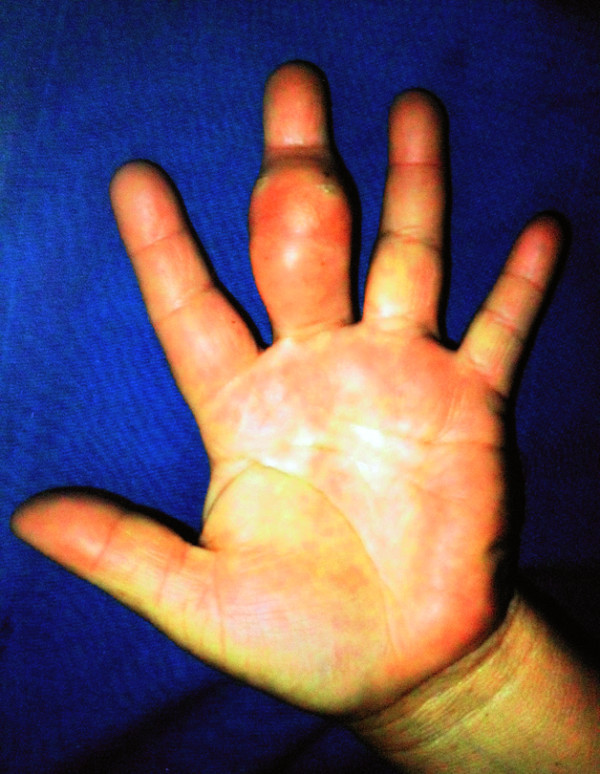
**Preoperative appearance of the patient showing a third left hand finger volar tumor.**

A 50-year-old otherwise healthy female arrived at our Plastic Surgery Department, with a one year history of a painless slow growing tumor on the volar aspect of the third finger of the left hand (Figure 1) that progressed to a limited interphalangeal joint movement prompting medical assistance.

The presenting lesion was a 50 × 20 mm subcutaneous mass in the proximal and middle phalanx, with a firm consistency but mobile over the underlying structures; she had discrete erythema and bluish skin color without palpable pulse, thrill or bruit. She presented limited movement in flexion of the interphalangeal joint and fingertip paresthesia. There was no local hyperthermia or manifestations of systemic disease.

The Roentgenogram showed a soft tissue swelling of low density over the middle phalanx, with no bone alteration.

**Figure 2:**
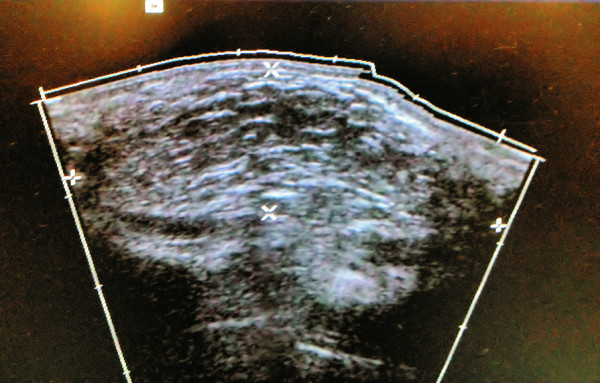
**Preoperative ultrasound scan of the tumor showed a well circumscribed isoechoic well defined mass.**

An ultrasound scan showed a well circumscribed isoechoic well defined ovoid mass, with a thin capsule and scarce septations, without alteration of the surrounding tissues (Figure 2).

**Figure 3:**
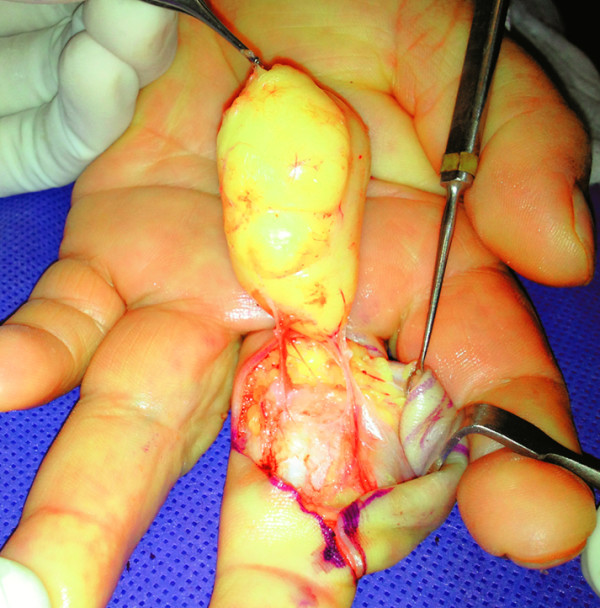
**Appearance of the tumor during the operation.**

It was decided to perform an excisional biopsy with a zealous and careful dissection, with clear identification of the neurovascular structures and tendons, the tumor was nourished by two pedicles that emerged from ulnar and radial digital bundle branches (Figure 3).

**Figure 4:**
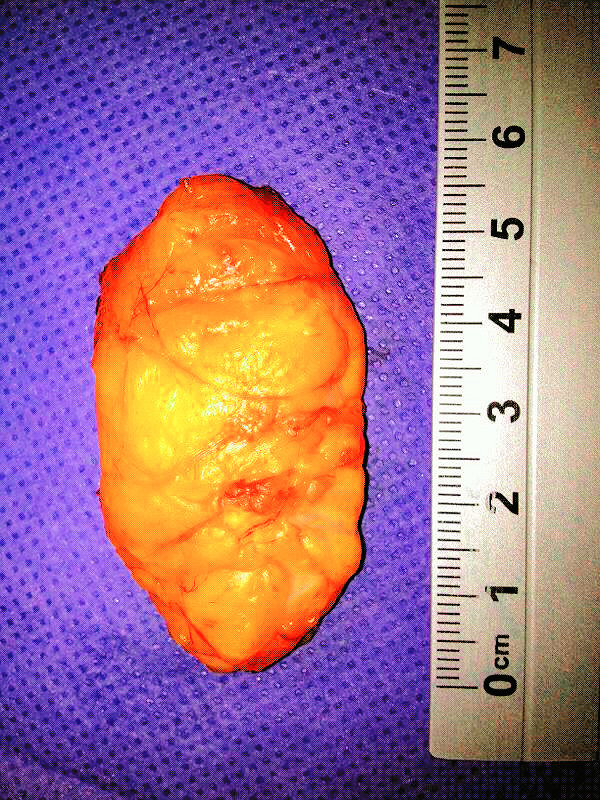
**Macroscopic appearance of the tumor with 53×25 mm dimensions.**

Pathology result reported as a lipoma that measured 53×25 mm with no neural component or malignant transformation (Figure 4).

She had an uneventful postoperative course, followed by complete movement and function of the finger. The reported fingertip paresthesia disappeared.

**Figure 5:**
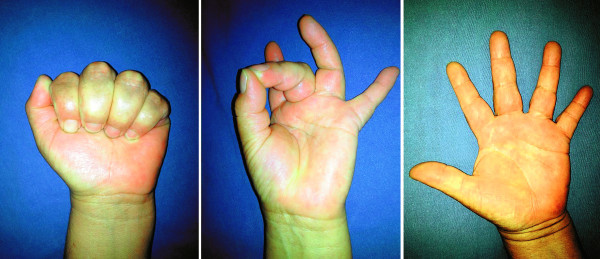
**Appearance of the hand after excisional biopsy.** Complete range of motion.

There was no evidence of recurrence of the tumor during the 12 months of postoperative follow-up with excellent range of motion and sensivity (Figure 5).

## Discussion

Lipomas account for approximately 16% of soft tissue mesenchymal tumors. They usually develop as a well-circumscribed, encapsulated mass with a doughy feel that is freely mobile underneath the skin. According to the 2002 World Health Organization’s committee for the Classification of Soft Tissue Tumors, they are categorized into 9 entities, including lipoma, lipomatosis, lipomatosis of nerve, lipoblastoma, angiolipoma, myolipoma of soft tissue, chondroid lipoma, spindle cell/pleomorphic lipoma and hibernoma. Benign lipomatous lesions affecting bone include intraosseous lipoma, parosteal lipoma and liposclerosing myxofibrous tumor. Benign lipomatous lesions may also affect joints and tendon sheaths, in a focal, or more commonly, a diffuse pattern. They are rarely encountered in the hand and with very less frequency in the digits. Its etiology is unknown, but multiple causative factors have been proposed which include genetic, traumatic and metabolic triggers. They appear mostly in the fifth and sixth decade. Presenting superficially or arising from the subfascial deep plane, in rare cases they can originate from juxta-articular regions or adjacent to the periostium as a parosteal lipoma or erode into the bone. Previous studies have defined a giant lipoma of the upper extremity as a mass larger than 5 cm (Cribb et al. 2005).

Lipoma in the hand typically presents with painless swelling and usually attains a large size by the time patients seek medical attention. Clinically, it has been described with grasping difficulties, decreased digital flexion and deviation of the fingers (Brand & Gelberman 1988). They can cause pain and distal sensory alterations with motor weakness. Accuracy of clinical evaluation reaches up to 85% in the superficial type (Kalisman & Dolich 1982).

Imaging studies are diagnostic in 71% of the cases, (Murphey et al. 2004) computed tomography and magnetic resonance have proved to be the gold standard (Horcajadas et al. 2003). In developing countries other less costly radiographic modalities are used in the preoperative evaluation, such as plain radiographs, where lipomas appear as an area of characteristic radiolucency referred to as a “water-clear density.” Ultrasound examination demonstrates a homogeneous and circumscribed hyperechoic or isoechoic area.

Surgical resection is the treatment of choice, it requires extensive dissection and mobilization of the neurovascular structures to accomplish success. Physician-patient relationship is key for the understanding of potential loss of function and other sequela. The main indication for their removal is the disturbance caused in hand functionality and cosmetic appearance (Higgs et al. 1993). The recurrence of this benign tumor is considered uncommon and reappearance is usually caused by technical surgical mishaps.

In this case, the patient sought for medical attention when movement limitation appeared with no other systemic association. It is clear that other neoplastic lesions and non-neoplastic lesions appear with clinical characteristics similar to those of a lipoma and should be considered as a differential diagnosis. When evaluating a tumor in the upper extremities the main concern is to rule out malignancy, which changes decision making and prognosis dramatically. Although lipoma is considered a rare entity in the hand, the surgeon should be aware of their existence due to its benign nature and favorable outcome before making radical decisions; as in this case, in which complete tumor resection lead to cure of the pathology with excellent cosmetic and function result.

In conclusion, lipomas of the digits are rare but benign tumors with a very limited risk of malignant transformation, they are associated with an excellent prognosis after successful excision; however, the preoperative evaluation requires a careful diagnostic workup due to its shared similarities to other pathologies.

## Consent

Written informed consent was obtained from patient for publication of this report and any accompanying images.
